# Review of "*Emerging Pests and Vector-Borne Diseases in Europe*" by Willem Takken and Bart G.J. Knols

**DOI:** 10.1186/1756-3305-1-30

**Published:** 2008-09-16

**Authors:** Domenico Otranto

**Affiliations:** 1 Department of Veterinary Public Health, University of Bari, 70010, Valenzano (Bari), Italy

## Review

VBDs, which constitute a global threat to human and animal health as well to livestock production, have been the subject of a number of publications ranging from periodicals to international conference proceedings and databases. "Emerging Pests and Vector-Borne Diseases in Europe" focuses on this subject and is the first volume in a new series of books on the "Ecology and Control of Vector-Borne Diseases" designed to provide readers with up-to-date information on different issues and current scientific priorities in vector ecology and VBDs. While the authors of future books in this series, which will be published annually, will deal with more specific subjects in the same field of research (i.e. Surveillance Methods of Vectors of Infectious Diseases, Mating Behaviours of Diseases Vectors, Recent Advances in the Control of Mosquitoes), "Emerging Pests and Vector-Borne Diseases in Europe" launches this European publishing venture with an overview of pests and VBDs. Given the difference of both approach and priorities in regard to the control and management of VBDs all over the world, the editorial decision to focus on Europe is certainly an original way of engaging with this topic. The chapters cover the interpretation of "emerging" and "re-emerging" and even the "resurgence" of many (but not all) VBDs and pests in Europe, concluding that this term cannot have an unambiguous meaning if not within the context of each subject.

Thus, the first section of this multi-authored book presents information on VBDs of (re-) emerging concern in Europe such as malaria, leishmaniasis, viral diseases (bluetongue, West Nile, Usutu, Chikungunya, Dengue), and tick-borne diseases causing encephalitis and Lyme disease. Particular attention has been paid to the reasons behind the spread of VBDs and to current forecasts about their diffusion. In this first part of the book, one might have expected to discover more about a number of tick-borne diseases (e.g. those caused by Rickettsiales, Bartonellaceae or haemoprotozoa), or about nematodes transmitted by biting insects. This is not the case.

The section on emerging pests in Europe includes remarkable chapters such as those on psoroptic mange (by R. Wall), on *Aedes albopictus *(by E.-J. Scholte and F. Schaffner), on bed bugs (by C. Boase) which, after a brief introduction to the biology of pests, discuss the risk of their spreading and the critical point for control/eradication measures. Other chapters in the same section would be better suited to an entomological textbook or to a pest-control manual. The last two sections include outstanding chapters which deal comprehensively with problems related to the surveillance, protection and control of VBDs and pests. Indeed, the chapter on monitoring systems for adult insects (by Yu Tong Qiu and colleagues) summarises the quantity of information available on commonly used insect monitoring tools, thus providing a very useful practical contribution to scientists working in the field. Again, readers are well catered for as regards personal protection against insect vectors (by N. O. Verhulst and colleagues): a judicious mixture of information and practical suggestions, which have been reviewed here with some originality. Similarly, the section dealing with nature conservation, wildlife management and human activities as drivers provides a satisfactory introduction (actually, as this is the final section, perhaps "epilogue" would be a better word!) to the risks of VBDs spreading throughout Europe. The chapters making up this section provide a good summary and discussion of the ecological and epidemiological aspects of the risk of spreading VBDs and pests in Europe.

In terms of general structure, the book is more akin to a collection of high quality review articles rather than to a work geared to one sole thread and a single editorial project. This is particularly noticeable in the first section of the book dealing with different VBDs, in which contributions do not stick to a similar style or structure and there has been no attempt to provide a short background to the pathogens, vectors or diseases. However, these concerns do not detract from the value of this scientific work which provides a critical discussion of the amount of data (mainly on the ecology) available with regard to some of the key pests and diseases affecting humans throughout Europe. A more in-depth discussion on the same topics with regard to both pet and livestock animals might well constitute a project for a further volume. As to general structure and content, the book is mainly aimed at scientists working in the field, although students interested in pests and VBDs would benefit from reading it. It would also be worth using this book as tracking and teaching material for seminars in Faculties of Human and Veterinary Medicine.

The publishers' ambition to produce a series of books dealing with different aspects of pests and VBDs has been achieved with this first volume, and we look forward to seeing further books in this series.

## List of abbreviations used

VBDs: Vector-borne diseases.

## Competing interests

The author declares that they have no competing interests.

